# Craniofacial and Dental Manifestations of Melnick–Needles Syndrome: Literature Review and Orthodontic Management

**DOI:** 10.1155/2018/5891024

**Published:** 2018-11-11

**Authors:** Dorota Kustrzycka, Marcin Mikulewicz, Anna Pelc, Piotr Kosior, Maciej Dobrzyński

**Affiliations:** ^1^Division of Facial Abnormalities, Department of Dentofacial Orthopedics and Orthodontics, Wroclaw Medical University, Krakowska 26, 50-425 Wroclaw, Poland; ^2^Department of Conservative Dentistry and Pedodontics, Wroclaw Medical University, Krakowska 26, 50-425 Wroclaw, Poland

## Abstract

The aim of this article was to present a characteristic clinical image of Melnick–Needles syndrome using an example of an 11.5-year-old female patient treated at the Facial Congenital Disorders Outpatient Clinic as well as to present the actual literature review of the surgical treatment. The patient was diagnosed with several characteristics typical for Melnick–Needles syndrome: single-sided hearing loss, malocclusion, and facial dysmorphism, among others. Due to malocclusion and facial dysmorphism, the patient with Melnick–Needles syndrome requires orthodontic treatment with surgical intervention. Mandibular distraction with fixed appliance treatment is a recommended treatment protocol.

## 1. Introduction

Melnick–Needles syndrome (MNS) (OMIM/Phenotype MIM number #309350, ICD 10- Q77.8), also named Melnick–Needles osseous dysplasia, is a very rare genetic disorder in which comorbid abnormalities in skeletal development are observed including the stomatognathic system. It was first reported by Melnick and Needles in 1966 and is the most serious disease of the otopalatodigital spectrum disorders [1].

Verloes et al. [2] reported that MNS is characterized by the most severe phenotypes in the spectrum of otopalatodigital syndromes and by the exclusive location of causal missense mutations in the exon 22 hotspot. The disease is caused by the mutation of gene FLNA, which encodes cytoskeleton protein filamin A [3]. MNS is one of the four syndromes caused by the mutation of the same gene; however, in this syndrome, it is related to the mutation in exon 22. Geneticists are able to refine clinical diagnosis according to the type and the location of FLNA mutations using molecular analyses [4]. Osteodysplasty of Melnick–Needles showed an increased content of collagen; its increased synthesis may be the expression of the sclerosing process [5]. Svejcar reported that the deficiency in alpha 1-chains may be the cause of the increase in cross-linking with a change in cleavage and extractability of collagen [5]. In most cases, this disease is coming into being *de novo*, and mostly it is inherited in an X-linked dominant manner. The Melnick–Needles syndrome appears more frequent in females, whereas male fetal sex mostly leads to miscarriage. Melnick–Needles syndrome is characterized by a short stature, underweight, face dysmorphism (prominent forehead, bilateral exophthalmos, fullness of the cheeks, and retrognathia), subluxation of certain joints, unusually long fingers and toes (flaring of the metaphyses of long bones), irregular constrictions in the ribs, and scoliosis. The amount and intensification of the resultant symptoms are often divergent from each other. Akin et al. [6] reported that Melnick–Needles syndrome can be associated with growth hormone deficiency. Severe mandibular hypoplasia can cause upper airway restriction, an increased incidence of sleep apnea, and pneumonias [7].

## 2. Materials and Methods

**Figure 1:**
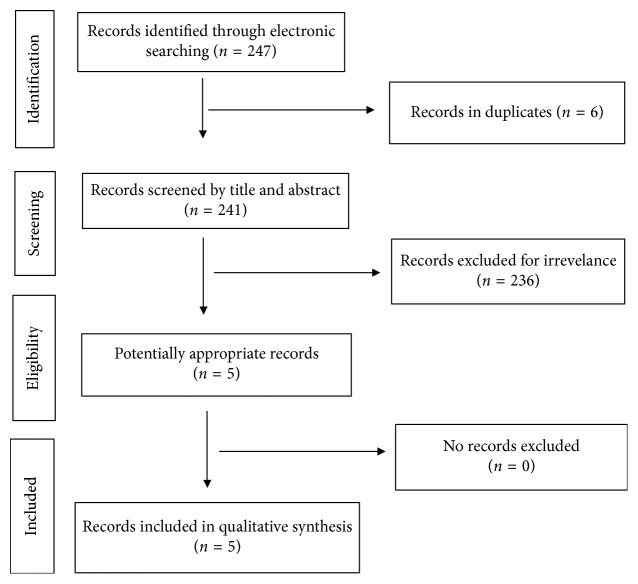
PRISMA flow diagram of study.

The review of the literature from the past 17 years (2000–2017) was done on 20 April 2017. Electronic bibliographic sources included PubMed and Scopus. The language of the articles was restricted to English. “Melnick–Needles,” “case report,” and “surgical” were the following keywords used for the search. 63 articles from Pubmed and 184 articles from Scopus were found. After identifying the duplicates and screening by title and abstract, five publications were selected (Figure 1). The including criteria referred to the surgical procedure in patients with Melnick–Needles syndrome (Table 1).

## 3. Aim

The aim of this article was to present a characteristic clinical image of Melnick–Needles syndrome using an example of an 11.5-year-old female patient and present actual literature review of surgical intervention among patients with Melnick–Needles syndrome.

### 3.1. Case Report

An 11.5-year-old female was referred to the Division of Facial Abnormalities at Wroclaw Medical University.

**Figure 2:**
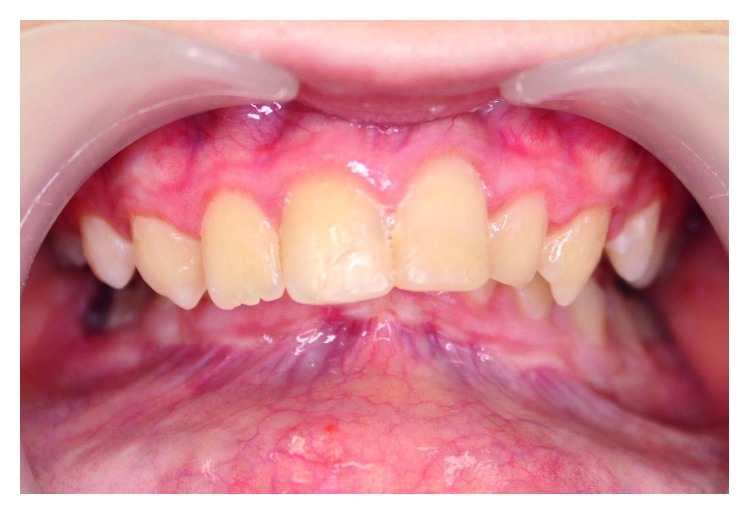
Intraoral photographs: frontal view.

**Figure 3:**
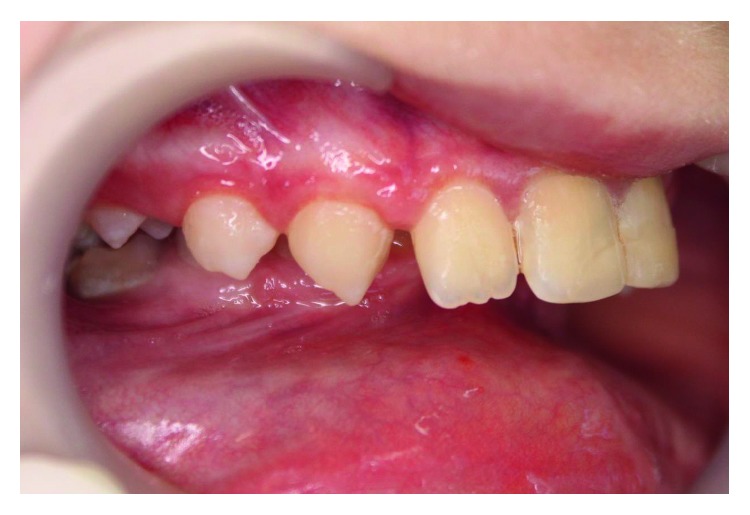
Intraoral photographs: right buccal.

**Figure 4:**
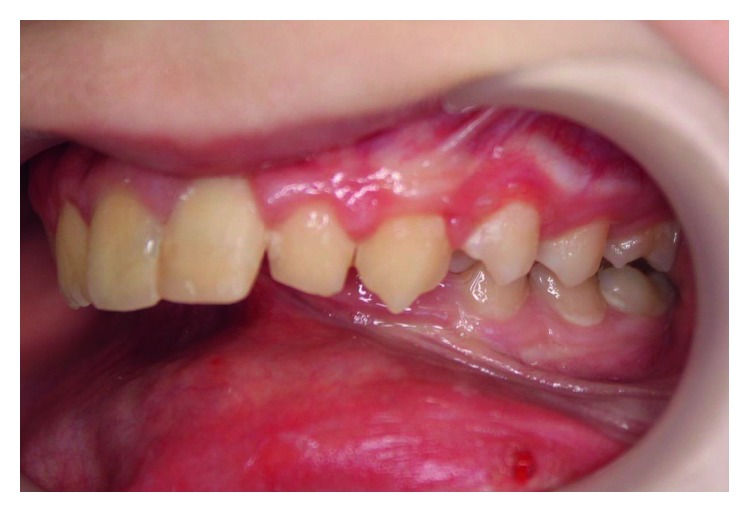
Intraoral photographs: left buccal.

**Figure 5:**
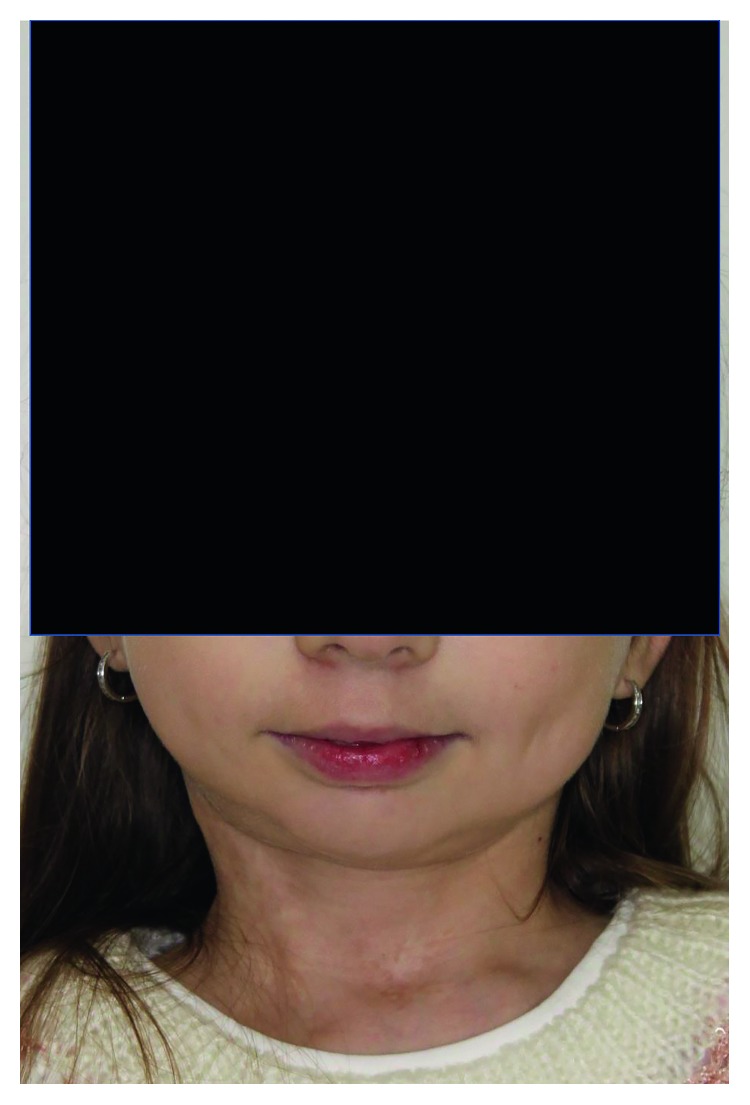
Extraoral photographs: face frontal.

**Figure 6:**
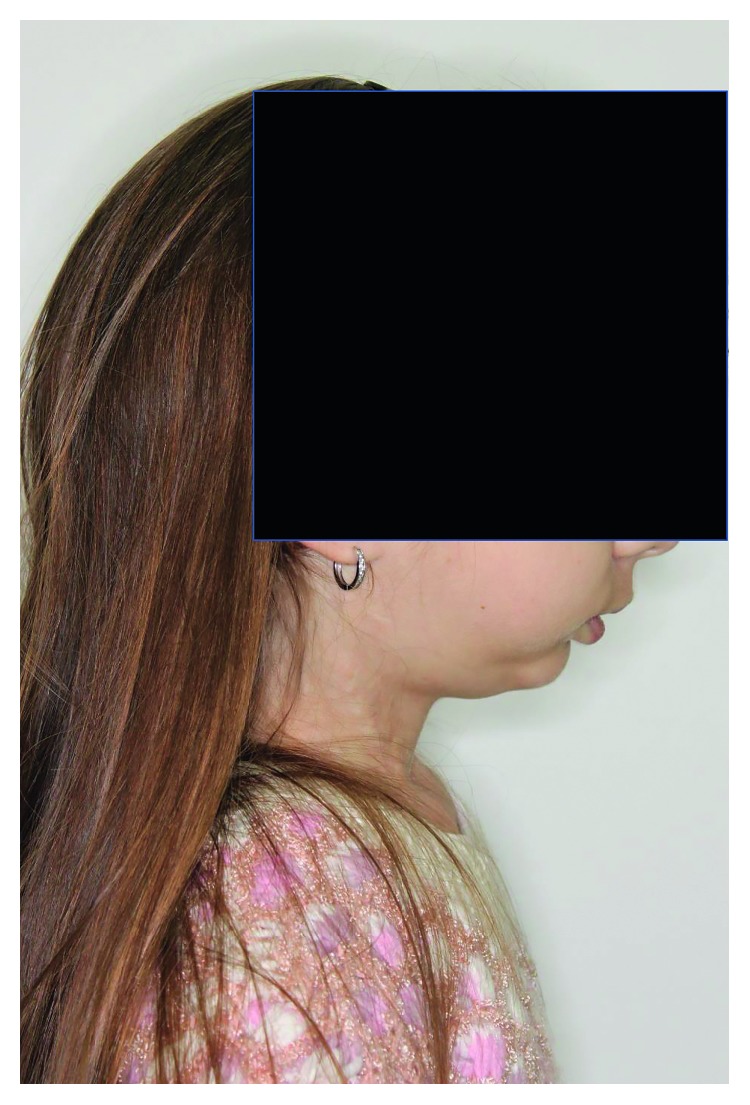
Extraoral photographs: profile.

**Figure 7:**
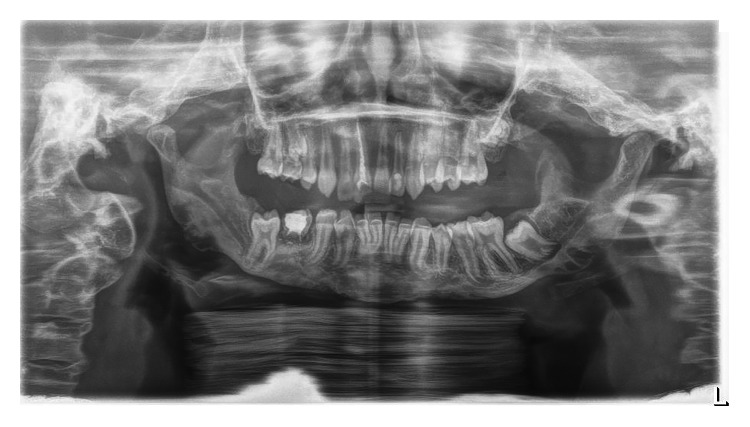
Panoramic radiograph.

**Figure 8:**
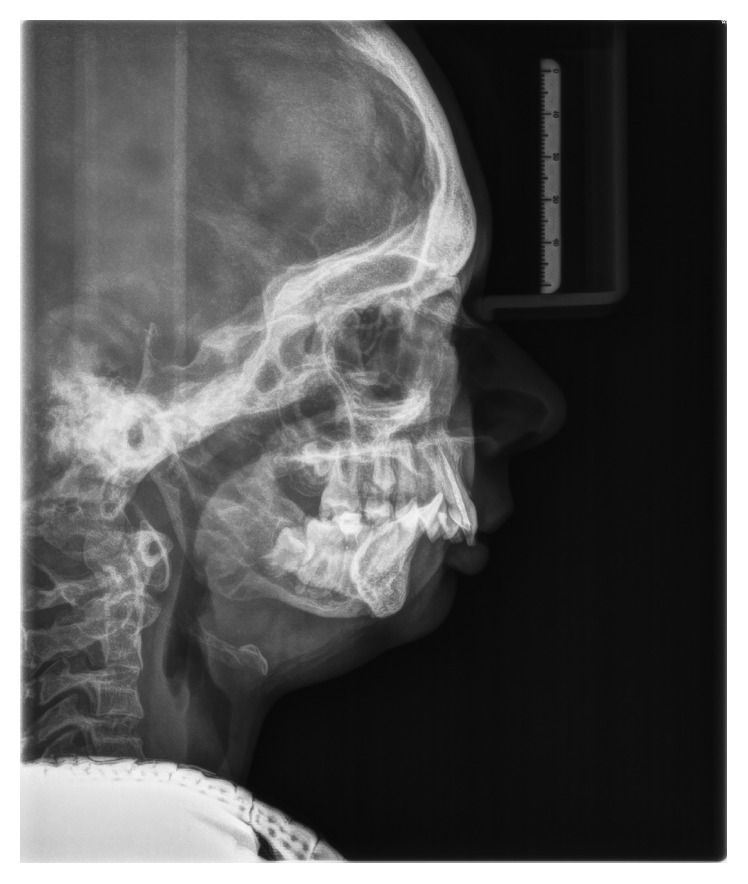
Cephalometric radiograph.

**Figure 9:**
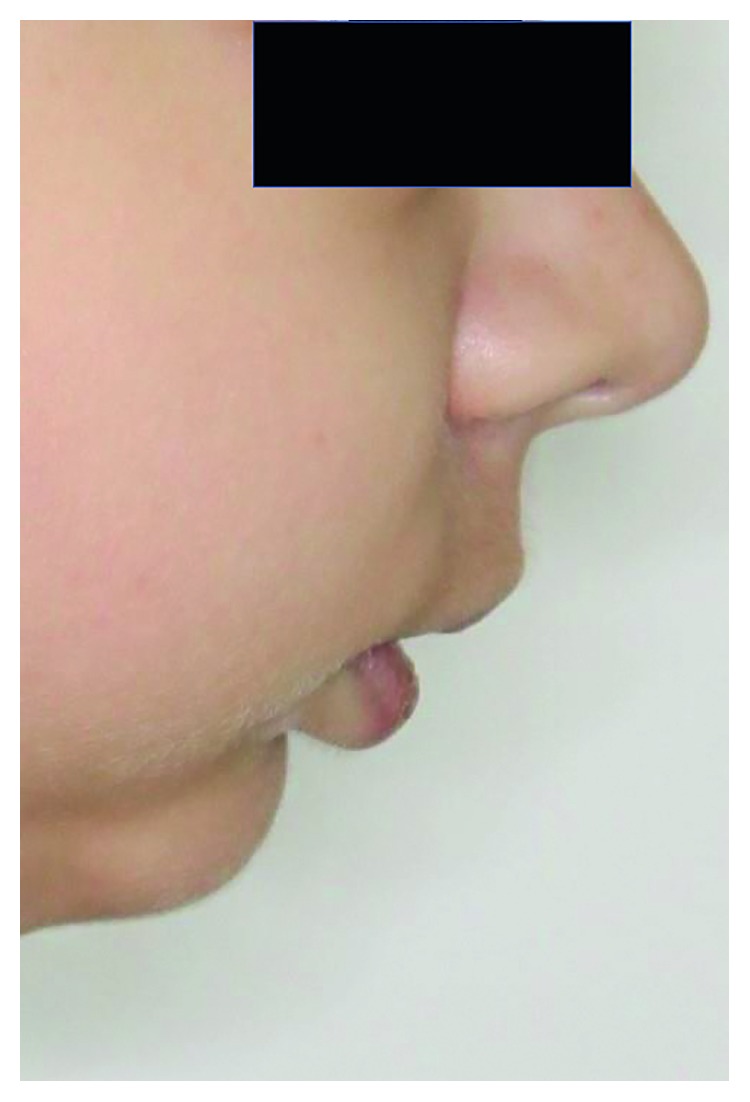
Profile photograph: specification of the oblique profile.

The girl was born of the first pregnancy without any eventful perinatal history. She weighed 3500 g. Based on the postnatal clinical examination, the additional digit in the left hand and torticollis and flexion contracture of the digits in the right hand with deformation of the right thumb were diagnosed. The genetic examination was done and the result showed mutation in the 22nd exon of gene FLNA (variant c.3956C) in heterozygous (what indicates the MNS). During the first year of the child's life, psychomotor development was insignificantly delayed. According to medical history, there were numerous abnormalities in the osteoarticular system and in the structure of the internal ear, facial dysmorphism, hypertelorism, bone loss in the frontal bone, and deformation of vertebral bodies of lumbar vertebrae and the child was underweight. The patient needed to remain under constant care of: pediatricians, audiologists, pulmonologists, and rehabilitation specialists. After clinical orthodontic examination (Figures 2–4), angle class II on the right and left side was diagnosed, overeruption (overjet 7.7 mm, overbite 12.7 mm) and facial dysmorphism (Figures 5 and 6): exophthalmos, hypertelorism, full cheeks, and prominent superciliary ridges were observed. Panoramic radiograph (Figure 7) demonstrated the absence of two right second tooth germs of the second molars (upper and lower), right second lower premolar, and all third molars. In place of the right lower second premolar, there was a persistent deciduous tooth 85. Results of the cephalometric analysis (Figure 8) indicated abnormalities in the following parameters: reduced mentolabialis sulcus angle, skeletal class II with proclination of the upper incisors (WITS 8.6 mm), retrognathic facial type–(Table 2), mandibular hypoplasia, which caused oblique retro face (Figure 9).

### 3.2. Differential Diagnosis

Melnick–Needles syndrome should be differentiated from Frank-Ter Haar syndrome—which exhibits characteristics similar to MNS. It differs clinically from MNS by presence of congenital glaucoma, and heart anomalies, brachycephaly, prominent forehead, protruding simple ears, and prominent coccyx are also regarded as important diagnostic signs [12]. Another syndrome that should be taken into account in the differential diagnosis is Shprintzen–Goldberg syndrome, which includes craniosynostosis, mental retardation, and marfanoid habitus. There appears to be a characteristic facies involving camptodactyly, downslanting palpebral fissures, inguinal or umbilical hernia, hypotonia, high-arched palate, and low-set posteriorly rotated ears [13]. Another differential syndrome is Pierre Robin sequence, that is characterized by glossoptosis and cleft of the secondary palate, which differ from the MNS [14]. Treacher Collins syndrome mainly differs by macrostomia, cleft palate, and antimongoloid slant of the eyes [15]. Crouzon syndrome should be differed by hypertelorism, parrot-beaked nose, short upper lip, hypoplastic maxilla, and a relative mandibular prognathism [16]. The most significant for the differential diagnosis is the genetic examination, which in our reported case indicates Melnick–Needles syndrome.

## 4. Discussion

Focusing on the stomatognathic system, in MNS, skeletal class II malocclusion is mostly recognized. In this instance, the most appropriate treatment protocol is to use mandibular distraction [17]. Molina et al. [11] presented their case report of successful treatment. After weighing the risks and benefits of the surgery, not every patient decides to undergo treatment with surgical intervention. Cephalometric analysis ascertained micrognathia. There are surgical (three-phase) and nonsurgical (two-phase) procedures. Both require an appliance that would extend the spatial dimension of the mandible during the growth spurt. The second phase consists of fixed appliance treatment. In the nonsurgical treatment, the aim is to eliminate enlarged overjet, protrude the mandible as much as possible to meliorate the patient's profile and to achieve occlusion on both sides. The surgical procedure consists of two phases mentioned above and the surgery. The fixed appliance treatment is divided into presurgical and postsurgical periods. The whole therapy lasts longer, as compared to the nonsurgical procedure, but the effects are more spectacular and stable [18]. The patient's parents have refused the future surgery due to the perioperative risk connected with the surgery as well as numerous postsurgical procedures aimed to correct any disorders of the patient's organs. This would involve a compromise treatment effect considering the lack of parental consent for the surgery. Now, the patient is treated with the use of the Schwarz plate, which is a removable appliance, in order to expand the maxilla and control the position of the incisors. This treatment phase provides proper preparation for the future fixed appliance treatment that enables more precise and faster movements of the teeth. Nevertheless, it requires very good oral hygiene, whereas the risk of insufficient hygiene among the group of 7–14-year-old children is increased. The fixed appliance will be placed on the upper and lower arch. The aim of this treatment phase will be to expand the lower arch and to eliminate the scissor bite in the posterior teeth, thereby giving the patient an appropriate occlusion. Overjet will stay enlarged because of too a small frontal dimension of the maxilla. Taking into account the decision of patient's parents concerning the refusal of the operation, the orthodontic treatment should, at least, improve the function of the stomatognathic system, which means it should create the occlusal plane. This provides appropriate mastication. The aesthetic aspect is minor but without the surgery, it is not possible to achieve the ideal profile.

## 5. Conclusions

Orthodontic treatment with surgical intervention—mandibular distraction—is a recommended treatment protocol for the Melnick–Needles syndrome with micrognathia.

Choosing the orthodontic treatment without a surgical procedure, the patient should be aware that the treatment outcome will not reestablish the appropriate function of the stomatognathic system.

## Figures and Tables

**Table 1:** Systematic review since 2000 (PubMed and Scopus): surgical procedures in patients with MNS.

Article	Publication type	No. of patients	Time of follow-up after surgery	Time of surgery	Surgery's type	Observations
Lykissas et al. [8]	Case report	2	8 years (25 years old)	17 years	Spine surgery	Well-maintained correction and no evidence of implant breakage
			5 years (18 years old	18 years		

Jung et al. [1]	Case report	1	8 months	18 years	Orthognatic surgery (BSSO)	Functional rehabilitation and aesthetic improvement have been achieved

Chen et al. [9]	Case report	1	No information	16 years	Orthognatic surgery (mandible)	Successful lengthening of mandible and full reconstruction of upper airway

Kelley et al. [10]	Case report	1	2 years (23 years old)	21 years	Orthognatic surgery (mandible)	The patient experienced complete resolution of symptoms and has been pain-free for more than 24 months

Molina et al. [11]	Case report	1	No information	No information	Orthognatic surgery	Occlusion had changed from a class II to a class III relationship. Snoring was eliminated. No need for tracheotomy in the future.

**Table 2:** Significant values of orthodontic cephalometric analysis.

Angle	Normal	Deviation	Patient value
SNA	82.0°	±3.0	76.8°
SNB	80.0°	±3.0	68.4°
ANB	2.0°	±2.0	8.4°
H	9.0°	±3.0	27.5°
1+ : 1−	133.0°	±8.0	117.5°
WITS	0.0°	2.0	8.6°
